# Establishment and Application of a Universal Coronavirus Screening Method Using MALDI-TOF Mass Spectrometry

**DOI:** 10.3389/fmicb.2017.01510

**Published:** 2017-08-09

**Authors:** Leshan Xiu, Chi Zhang, Zhiqiang Wu, Junping Peng

**Affiliations:** Ministry of Health Key Laboratory of Systems Biology of Pathogens, Institute of Pathogen Biology, Chinese Academy of Medical Sciences & Peking Union Medical College Beijing, China

**Keywords:** coronavirus, human coronavirus, MALDI-TOF mass spectrometry, respiratory infection, detection

## Abstract

There are four human coronaviruses (HCoVs), distributed worldwide, that are associated with a range of respiratory symptoms. The discovery of severe acute respiratory syndrome (SARS)-CoV and Middle East respiratory syndrome (MERS)-CoV shows that HCoVs pose a significant threat to human health. Our work aims to develop a sensitive method (mCoV-MS) which can not only identify known HCoVs accurately, but also have the ability to provide clues for the emerging HCoVs. The method was performed using a MassARRAY matrix-assisted laser desorption/ionization time-of-flight mass spectrometry (MALDI-TOF MS) system. We developed a 17-plex analysis to detect six HCoVs in Panel A and another 17-plex analysis to detect *Alphacoronavirus* and *Betacoronavirus* in Panel B. All tested primers and probes for the mCoV-MS method were effective, with no cross-reactivity observed with other common respiratory viruses. To confirm the usefulness of the mCoV-MS method we screened 384 pharyngeal and/or anal swab samples collected from bats/rodents, and 131 nasal and throat swabs from human patients. The results showed good concordance with the results of metagenomic analysis or PCR-sequencing. The validation test showed mCoV-MS method can detect potentially pathogenic CoVs in *Alphacoronavirus* and *Betacoronavirus* and provide convincingly phylogenetic evidences about unknown CoVs. The mCoV-MS method is a sensitive assay that is relatively simple to carry out. We propose that this method be used to complement next generation sequencing technology for large-scale screening studies.

## Introduction

Coronaviruses (CoVs) are large, enveloped, positive-sense RNA viruses that can cause respiratory, enteric, hepatic, and neurological diseases in a range of animals, including humans (Lai et al., 2007). According to the latest criteria of International Committee on Taxonomy of Viruses, CoVs are classified into four genera, including *Alphacoronavirus, Betacoronavirus, Deltacoronavirus*, and *Gammacoronavirus*. There are six human coronaviruses (HCoVs), HCoV-229E, HCoV-OC43, HCoV-NL63, HCoV-HKU1, severe acute respiratory syndrome coronavirus (SARS-CoV), and Middle East respiratory syndrome coronavirus (MERS-CoV) which belong to *Alphacoronavirus* and *Betacoronavirus*. HCoV-229E, HCoV-OC43, HCoV-NL63, and HCoV-HKU1 are usually associated with relatively mild respiratory symptoms and the detection rate ranges from 0.3 to 4.5% in respiratory specimens according to different studies (Gaunt et al., 2010; Gadsby et al., 2016; Trombetta et al., 2016). In 2003, SARS emerged from China as a rapidly spreading respiratory illness with a fatality rate close to 10%. The SARS-CoV was found to be a lineage B *Betacoronavirus* and was confirmed as the etiological agent of SARS (Drosten et al., 2003; Ksiazek et al., 2003; Lee et al., 2003; Peiris et al., 2003). In 2012, MERS-CoV was detected in humans, which is a lineage C *Betacoronavirus* that differs from other HCoVs. This virus has caused severe acute respiratory infection (SARI) in patients with a fatality rate close to 40% and led to a new awareness of the medical importance of HCoVs (van Boheemen et al., 2012; Zaki et al., 2012; de Groot et al., 2013). As of 15 June 2017, there were 2015 laboratory confirmed cases of MERS-CoV infection and 703 deaths in 27 countries (http://www.who.int/emergencies/mers-cov/en/). The emergence of SARS-CoV and MERS-CoV suggests that highly pathogenic HCoVs remain a key threat to human health. Because of the severity and communicability of SARS and SARI, rapid and sensitive diagnostic methods are essential for the timely implementation of effective infection control measures to prevent further transmission. Millions of pilgrims from across the globe perform the Hajj annually, increasing the risk of global transmission of MERS-CoV (Sharif-Yakan and Kanj, 2014). The large number of tourists traveling to the Middle East also increases the risk of transmitting MERS-CoV to other areas. Thus, it is important to develop a high-throughput and sensitive method that can detect HCoVs, especially MERS-CoV.

Isolation of virus from clinical specimens by cell culture is regarded as the gold standard for laboratory diagnosis of respiratory virus infection. However, for HCoVs, attempting to isolate virus has long turnaround time and some HCoVs are difficult to culture, which makes the detection results lack timeliness. Compared with cell culture, molecular tests, such as reverse transcription polymerase chain reaction (RT-PCR), nested RT-PCR, and quantitative real-time RT-PCR assays (qPCR) are more rapid, and have superior sensitivity (van der Hoek et al., 2004; Bellau-Pujol et al., 2005; Vijgen et al., 2005; Vabret et al., 2006; Gaunt et al., 2010). To date, molecular tests are accepted worldwide as an important method for diagnosing HCoV infection. However, most of the current molecular methods target a single HCoV gene, which limits their application because of the mutations that can occur in HCoV genomes. Thus, false negative results can be returned because they are only targeting one gene.

In view of the lessons we learned from MERS and SARS outbreak, CoVs have the ability of interspecies transmission and may emerge as novel pathogens (Chan et al., 2013; Ge et al., 2013). Therefore, an ideal diagnosis method should not only identify known HCoVs accurately, but also have the ability to provide clues for the emerging HCoVs. However, most of the current molecular assays only target some of HCoVs, which may cause a false negative result when facing novel HCoV infections. In this study, we designed and evaluated a multiplexed CoVs test, mCoV-MS, which can detect 6 known HCoVs and have the potential to discover new HCoVs that haven't been clearly described yet. Our new method consists of two panels. Panel A is designed to cover six known HCoVs. Panel B is designed to cover all the known CoVs in *Alphacoronavirus* and *Betacoronavirus* which is developed to expand our detection spectrum. Our method is based on the MassARRAY matrix-assisted laser desorption ionization-time of flight (MALDI-TOF) mass spectrometry (MS) system (Agena Bioscience, Inc., San Diego, CA, USA), which has been used for pathogen detection successfully, including common respiratory viruses, human papillomavirus, human polyomavirus, human enterovirus and *Staphylococcus aureus* (Yang et al., 2005; Soderlund-Strand et al., 2008; Basu et al., 2011; Syrmis et al., 2011; Li et al., 2013, 2015; Peng et al., 2013a,b, 2014, 2016b,a; Zhang et al., 2015). Likewise, the mCoV-MS method uses multiplex PCR conjugated MALDI-TOF MS technology to achieve high-throughput CoVs detection. The method was divided into three stages: multiplex PCR, primer extension and MALDI-TOF MS identification. Briefly, all the targets are amplified via multiplexed PCR followed by incubation with shrimp alkaline phosphatase (SAP) to dephosphorylate any unincorporated dNTPs. In primer extension stage, site-specific short oligonucleotide primers will bind the respective amplicons and are extended a single base, as the substrate used in this reaction is ddNTPs. Finally, MALDI-TOF MS is used to measure the masses of extended primers accurately, thus receiving a positive or negative result for every target (Oeth et al., 2009).

## Materials and methods

### Design of the mCoV-MS method

Representative HCoV strains used in the design of the mCoV-MS method are shown in Supplementary Table 1. The CoV genome sequences used for primer and extension probe design were obtained from GenBank (http://www.ncbi.nlm.nih.gov/genbank/). Specific primers and extension probes (Supplementary Table 2) used in this study were designed using Assay Design v4.0 (Agena Bioscience, Inc.). In this study, we developed a 17-plex analysis to detect six HCoVs in Panel A and another 17-plex analysis to detect *Alphacoronavirus* and *Betacoronavirus in* Panel B. Panel A: including specific primers and extension probes targeting the RNA-dependent RNA polymerase (*RdRp*) and nucleocapsid (*N*) genes of six HCoVs, and ribonuclease P (as an internal control). In addition, for SARS-CoV and MERS-CoV, we also designed primers and extension probes targeting the *ORF1b* gene and regions upstream of the E gene (*upE*). Panel B: including specific primers and extension probes targeting *RdRp* gene of CoVs in *Alphacoronavirus* and *Betacoronavirus*.

### Experiment procedures of mCoV-MS method

The primary PCR and iPLEX reaction of the mCoV-MS method was performed in a 384-plate by using a ProFlex PCR system (Applied Biosystems, USA) according to our modified protocol as previously described (Peng et al., 2013a). The PCR mixes (5 μl) contained 2 μl template and final concentrations of 500 μM nucleotide mix (dATP, dGTP, dCTP, and dUTP), 100 nM primer mix, 1 U DNA polymerase enzyme, 2 mM MgCl_2_, and 0.2 U uracil-DNA glycosylase. The reaction conditions of primary PCR were: 45°C for 2 min; a denaturing step of 2 min at 95°C followed by 45 cycles of 95°C for 30 s, 56°C for 30 s and 72°C for 1 min; and then a final extension step at 72°C for 5 min. Sterile water was used as negative control. Then SAP was used to dephosphorylate the primary PCR mixes as follows: 37°C for 40 min and then 85°C for 5 min. After SAP treatment, the iPLEX reaction (single-base extension reaction) mix which included 0.2 μl terminator mix, 0.94 μl extension probe mix, 0.2 μl iPLEX Pro buffer, and 0.041 μl ThermoSequenase enzyme was added. The iPLEX reaction, desalting and sample dispensing were performed according to standard procedures (Agena Bioscience, Inc.). All data were acquired and analyzed using Typer software, v4.0.3 (Agena Bioscience, Inc.).

### Test of the mCoV-MS method

We used the plasmids containing nearly the full-length sequence of target genes of HCoVs to determine the analytical sensitivity of the mCoV-MS method. The plasmids were diluted to a series of concentrations of 1, 10, 100 1,000, and 10,000 copies per reaction. The test of each plasmid concentration was performed in triplicate.

All samples used in this study were obtained from collections at our institute. We isolated total RNA and DNA from all samples using the Qiagen DNA/RNA isolation kits (Qiagen, Germany) according to the manufacturer's instructions. To evaluate the specificity of the mCoV-MS method, we used 32 samples that had previously been confirmed positive for HCoV (Ren et al., 2009; Zhang et al., 2014), and two bat SARS-like coronavirus isolated from bats (Yang et al., 2013). A panel of common respiratory viruses (Ren et al., 2009; *n* = 110) was also used to determine the specificity of the mCoV-MS method (Table 1).

**Table 1:** Confirmed clinical samples used in the study.

**Virus name**	**Numbers**
**CORONAVIRUS**	
HCoV-229E	7
HCoV-OC43	8
HCoV-NL63	8
HCoV-HKU1	8
MERS-CoV	1
Bat SARS-like coronavirus	2
Total	34
**OTHER COMMON RESPIRATORY VIRUSES**	
Human enterovirus 71	10
Coxsackievirus A16	12
Human rhinovirus	6
Human adenovirus	16
Influenza virus A H1N1	5
Influenza virus A H1N1 pdm09	4
Influenza virus A H3N2	6
Influenza virus B	2
Human metapneumovirus A	3
Human metapneumovirus B	2
Respiratory syncytial virus A	5
Respiratory syncytial virus B	5
Human parainfluenza virus 1	10
Human parainfluenza virus 2	2
Human parainfluenza virus 3	2
Human parainfluenza virus 4	2
Human bocavirus 1	5
Human bocavirus 2	1
Human bocavirus 3	1
WU polyomavirus	6
KI polyomavirus	5
Total	110

Potential biohazardous materials were handled in required levels of biosafety laboratories. All experiments (collection of samples, sample handing, operator training and protection, etc.) were carried out according to the approved biosafety standard guidelines set by The Institute of Pathogen Biology, Chinese Academy of Medical Sciences & Peking Union Medical College.

### Application of the mCoV-MS method

Further testing of the mCoV-MS method involved 384 pharyngeal and/or anal swab samples collected from bats (*n* = 352) or rodents (*n* = 32) which were part of our bat and rodent virome project. *Alphacoronavirus* and *Betacoronavirus* in 384 samples of bats and rodents were also screened using a RT-PCR of the *RdRp* gene of CoVs (Lau et al., 2012). For comparison, the next generation sequencing technology (NGST) results of 352 bat samples in another study were also included (Wu et al., 2016).

We screened 131 nasal and throat swabs obtained from patients for HCoVs using the mCoV-MS method. The results were compared with those of other methods as described follows. Four common HCoVs were also screened by using PCR or nested PCR assays, followed by sequencing analysis according to previously reported methods (van der Hoek et al., 2004; Bellau-Pujol et al., 2005; Vabret et al., 2006). To screen for SARS-CoV and SARS-like CoV, we used the method described by the World Health Organization (http://www.who.int/csr/sars/primers/en/). To screen for MERS-CoV in samples, we used a qPCR assay developed by Corman et al. (2012a,b).

### Statistical analysis

We tested the differences between the detection rates of the two different methods using the χ^2^ test. A *P* < 0.05 was considered statistically significant.

## Results

### Performance of the mCoV-MS method

**Figure 1:**
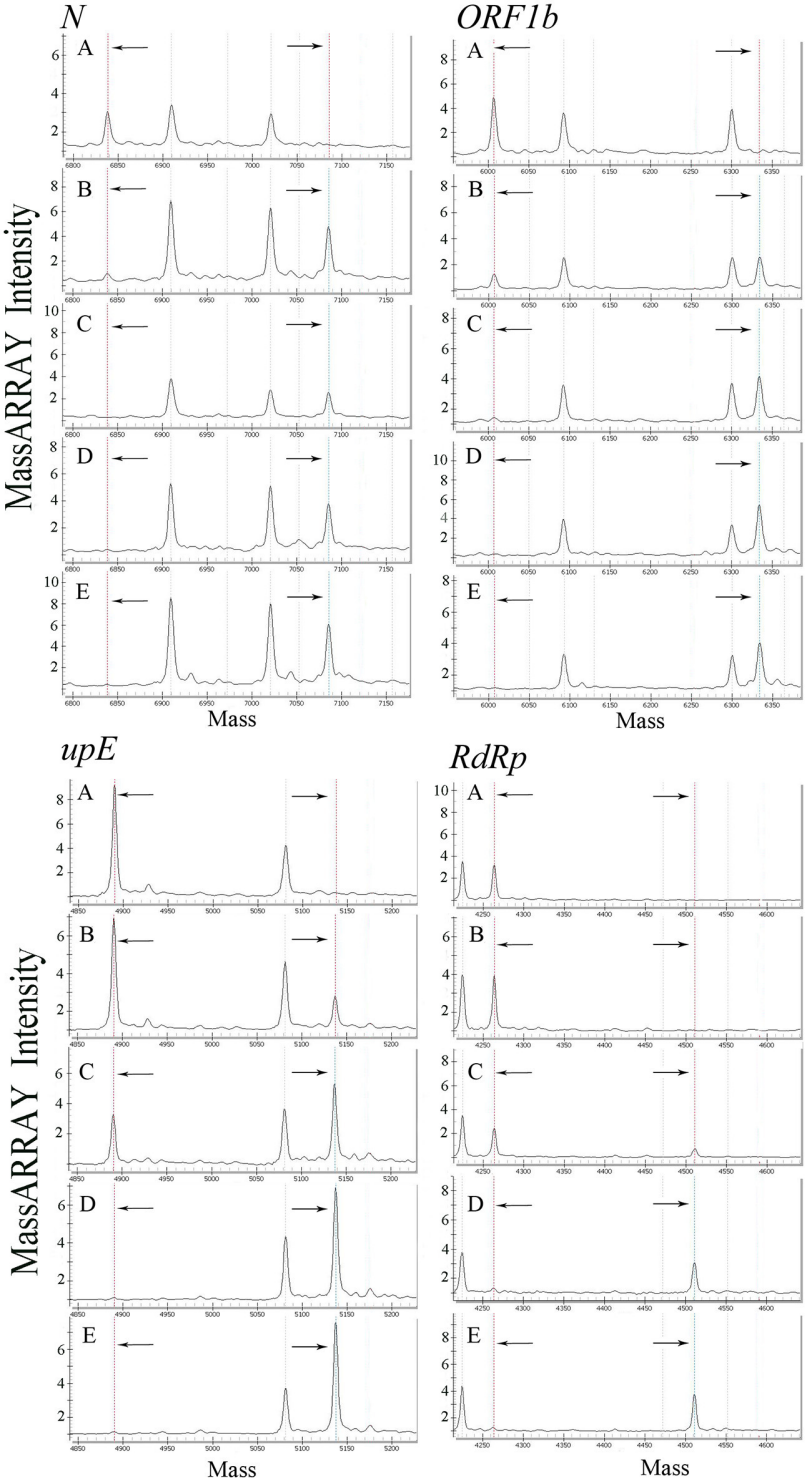
Analysis of a dilution series of MERS-CoV plasmids using mCoV-MS method. **(A)** 1 copy/reaction, **(B)** 10 copies/reaction, **(C)** 100 copies/reaction, **(D)** 1,000 copies/reaction, **(E)** 10,000 copies/reaction. In the mass spectrometry mass spectra, the dotted lines in the left and the dotted lines in the right represent the unextended primers and the extended primers of the assay, respectively.

All primers and probes used in the mCoV-MS method were evaluated using BLASTN searches against the non-redundant nucleotide database of the National Center for Biotechnology Information. Panel A and B test can be carried out simultaneously or separately according to actual needs. Sensitivity tests showed that the panel A of mCoV-MS method can accurately detected all six targeted HCoVs. The limit of detection of the mCoV-MS method was ~10–100 copies per assay panel (Table 2). For example, for the MERS-CoV panels, we were able to detect 10 copies of *N, ORF1b* and *upE*, and 100 copies of *RdRp* (Figure 1). As expected, negative controls yielded no positive results for the mCoV-MS method.

**Table 2:** The detection limits of the mCoV-MS method.

**Assays**	**Detection limit (copies/reaction)**
MERS-CoV_RdRp	100
MERS-CoV _N	10
MERS-CoV_ORF1b	10
MERS-CoV_upE	10
SARS-CoV_RdRp	10
SARS-CoV_N	10
SARS-CoV_ORF1b	100
SARS-CoV_upE	10
229E_RdRp	100
229E_N	100
NL63_RdRp	100
NL63_N	10
OC43_RdRp	100
OC43_N	10
HKU1_RdRp	100
HKU1_N	10

We used 32 HCoV-positive samples to evaluate the common HCoV assays and MERS-CoV assays, and two SARS-like CoV isolates to evaluate the SARS-CoV assays (Table 1). Our results showed that all combinations of primers and probes were effective (Supplementary Figures S1–S5), with no observed cross-reactivity with other HCoVs.

A panel of common respiratory viruses (influenza A virus, influenza B virus, adenovirus, respiratory syncytial virus A, respiratory syncytial virus B, parainfluenza virus types 1–4, human metapneumovirus A, human metapneumovirus B, rhinovirus, human enterovirus 71, coxsackievirus A16, human bocavirus 1–3, WU polyomavirus, and KI polyomavirus) were also used in this study (Table 1). Our results clearly showed no cross-reactivity with other common respiratory viruses.

### To screen bat and rodent samples using the mCoV-MS method

We used 352 pools of bat pharyngeal and anal swab samples to validate the performance of mCoV-MS. These samples were collected in 22 provinces which is a part of our bat virome project (Wu et al., 2016). Among the 352 pools, 20 (5.68%) were tested positive by our method. Seventeen pools (4.83%) were identified by panel A assays as SARS-CoV, which showed good concordance with the results of SARS-like CoVs by means of metagenomic analysis (Wu et al., 2016; Table 3). Six of the 17 (35.3%) pools were also confirmed as lineage-B beta-CoV by panel B assays. We found that these samples obtained two or more positive results by panel A assays. It is obvious that multiple target using in panel A avoid the false negative results. Five of the 17 (29.4%) pools were confirmed as lineage-B beta-CoV by PCR-sequencing method. It seemed that the mCoV-MS method were more sensitive than PCR method (*P* = 0.01 by χ^2^ test). Interestingly, all the 17 pools obtained SARS-*upE*-positive results, while only 8 (47.1%) of them were detected by the panel B assays or PCR method targeting CoV's *RdRp* gene. Our results are consistent with the earlier research demonstrating that *upE* assay is the most sensitive test for coronavirus detection (Corman et al., 2012a; Chan et al., 2015). The remaining three pools (0.9%) were identified as *Alphacoronavirus* by panel B assays and were ascertained by PCR-sequencing method.

**Table 3:** CoV positive samples from bats.

**ID**	**Source**	**Species**	**mCoV-MS results**		**PCR results**	Virome analysis results^*^	
			**Panel A**	**Panel B**		**NGST results**	**full-length sequence obtained**
B1	Fu Jian	Hipposideros armiger	*SARS_upE*	–	–	lineage-B beta-CoV	–
B2	Fu Jian	Rhinolophus lepidus	*SARS_upE*	–	–	lineage-B beta-CoV	–
B3	Guang Xi	Rhinolophus sinicus	*SARS_N, ORF1b, upE*	lineage-B beta-CoV	lineage-B beta-CoV	lineage-B beta-CoV	KJ473815
B4	He Bei	Rhinolophus ferrumequinum	*SARS_upE*	–	–	lineage-B beta-CoV	KJ473812
B5	He Nan	Rhinolophus ferrumequinum	*SARS_upE*	–	–	lineage-B beta-CoV	KJ473817
B6	Hu Bei	Myotis daubentonii	*SARS_N, upE*	lineage-B beta-CoV	–	lineage-B beta-CoV	KJ473818
B7	Hu Bei	Rhinolophus ferrumequinum	*SARS_upE*	–	–	lineage-B beta-CoV	KJ473819
B8	Ji Lin	Rhinolophus ferrumequinum	*SARS_upE*	–	lineage-B beta-CoV	lineage-B beta-CoV	KJ473811
B9	Liao Ning	Rhinolophus ferrumequinum	*SARS_upE*	–	lineage-B beta-CoV	lineage-B beta-CoV	–
B10	Jiang Xi	Myotis ricketti	*SARS_upE*	–	–	lineage-B beta-CoV	–
B11	Ning Xia	Plecotus auritus	*SARS_upE*	–	–	lineage-B beta-CoV	–
B12	Shaan Xi	Rhinolophus ferrumequinum	*SARS_upE*	–	–	lineage-B beta-CoV	KJ473813
B13	Shaan Xi	Rhinolophus pusillus	*SARS_N, ORF1b, upE*	lineage-B beta-CoV	lineage-B beta-CoV	lineage-B beta-CoV	JX993987
B14	Shaan Xi	Miniopterus schreibersii	*SARS_upE*	–	–	lineage-B beta-CoV	–
B15	Yun Nan	Cynopterus sphinx	*SARS_N, ORF1b, upE*	lineage-B beta-CoV	–	lineage-B beta-CoV	–
B16	Zhe Jiang	Rhinolophus sinicus	*SARS_N, upE*	lineage-B beta-CoV	–	lineage-B beta-CoV	–
B17	Zhe Jiang	Hipposideros pratti	*SARS_N, upE*	lineage-B beta-CoV	lineage-B beta-CoV	lineage-B beta-CoV	KF636752
B18	Guang Dong	Miniopterus schreibersii	–	Alpha-CoV	Alpha-CoV	Alpha-CoV	KJ473797
B19	He Nan	Miniopterus fuliginosus	–	Alpha-CoV	Alpha-CoV	Alpha-CoV	KJ473800
B20	Jiang Xi	Miniopterus fuliginosus	–	Alpha-CoV	Alpha-CoV	Alpha-CoV	KJ473796

* *Wu et al. (2016)*.

Another 32 rodent pharyngeal and anal swab pools collected in eight provinces across China were also screened by our novel method. A total of 4 (12.5%) pools were classified as lineage-A *Betacoronavirus* by panel B assays and PCR-sequencing verified two of them (Table 4).

**Table 4:** CoV positive samples from rodents.

**ID**	**Source**	**Species**	**mCoV-MS results**		**PCR results**
			**Panel A**	**Panel B**	
R01	Hainan	*Rattus rattus*	–	lineage-A beta-CoV	–
R02	Guangdong	*Mus musculus*	–	lineage-A beta-CoV	lineage-A beta-CoV
R03	Xinjiang	*Microtus gregalis*	–	lineage-A beta-CoV	–
R04	Guizhou	*Apodemus agrarius, Niviventer niviventer*	–	lineage-A beta-CoV	lineage-A beta-CoV

### To screen clinical samples using the mCoV-MS method

Using the mCoV-MS method, we analyzed 131 nasal and throat swabs. The mCoV-MS method detected HCoVs in 22.14% (29/131) of samples. This result was identical to that when we used a combination of PCR and sequencing techniques. We detected HCoV-OC43, HCoV-229E, HCoV-NL63, and HCoV-HKU1 in 9.9% (13/131), 6.9% (9/131), 3.1% (4/131) and 2.3% (3/131) of specimens, respectively. SARS-CoV and MERS-CoV were not detected in any of these samples.

## Discussion

The emergence of SARS-CoV and MERS-CoV has tested our ability to design new diagnostic methods for their rapid detection. Lesson from SARS showed that we were not adequately prepared for the first pandemic of the twenty-first century (de Wit et al., 2016). It took several months to confirm the causative agent as SARS-CoV. Fortunately, advances in molecular technology, especially NGST, allows us to timely and accurately identify MERS-CoV (Zaki et al., 2012). It is very important that these viruses are rapidly and accurately identified in order to assist in commencing an appropriate treatment regimen for patients. The emergence of MERS-CoV has once again highlighted the impact of HCoVs on human health (Chan et al., 2015). Several studies have shown that HCoVs likely originate from wild animal hosts, such as bats (Calisher et al., 2006; Wang et al., 2006; Woo et al., 2012). Shi and colleagues found a SARS-like CoV in Chinese horseshoe bats, suggesting that bats are the natural reservoir of SARS-CoV, and palm civets are an incidental host (Li et al., 2005; Ge et al., 2013). Huynh et al. found that HCoV-NL63 might share common ancestry with *Alphacoronaviruses* of the North American tricolored bat (Huynh et al., 2012). Several groups have shown that MERS-CoV also likely originates in bats (Annan et al., 2013; Ithete et al., 2013; Memish et al., 2013). Based on neutralizing serum antibody tests, Reusken et al. showed that MERS-CoV, or a related virus, has infected camels (Reusken et al., 2013). A multidisciplinary team of virologists isolated MERS-CoV from dromedary camels (Haagmans et al., 2014). Alagaili et al. also showed that MERS-CoV has been circulating in camels for nearly 20 years; however there is no evidence of MERS-CoV infection in domestic goats or sheep (Alagaili et al., 2014). The identification of SARS-CoV and MERS-CoV indicates that viral cross-species transmission is a real threat to human health (Chan et al., 2013; de Wit et al., 2016). Recently, Menachery et al. suggested that a cluster of SARS-like CoV circulating in bat populations shows potential for human emergence (Menachery et al., 2015). It is possible there are additional CoVs circulating in the wild that could infect humans. To confirm this, a large-scale study screening samples from various wild animals all over the world needs to be conducted. However, it is possible that false negative results would be seen using conventional detection methods that target one gene, because of the genetic diversity of HCoVs.

In clinical laboratories, it has been shown that qPCR assays are sensitive detection methods, which are also quantitative (Vijgen et al., 2005; Gaunt et al., 2010). Most qPCR-based methods target a single gene of a virus. Gaunt et al. performed a large-scale screening of four common HCoVs using a 4-plex qPCR assay, thereby significantly improving HCoV diagnosis (Gaunt et al., 2010). It has been shown that qPCR assays have played an important role in the detection and identification of MERS-CoV. Multiple methods targeting different genes of MERS-CoV have been developed and used to detect MERS-CoV. Corman et al. successfully developed several qPCR assays targeting *upE, ORF1b* and *ORF1a* (Corman et al., 2012a,b). In another study, Lu et al. developed a multiplex qPCR assay targeting the *N* gene and the *upE* region (Lu et al., 2014). However, the availability of fluorescent dyes limits the multiplexing capacity of their qPCR assay. The mCoV-MS method we developed targets two or four genes, making it a powerful method for screening samples from humans and other animals. Our results also showed that multiple target using in panel A avoid the false negative results. The validation test showed our method can target potentially pathogenic CoVs in *Alphacoronavirus* and *Betacoronavirus* and provide convincingly phylogenetic evidences about unknown CoVs. Thus, our method is an effective way to expand CoVs detection spectrum.

NGST is able to generate a large amount of data by random sequencing, and has been widely used in pathogen detection (Haagmans et al., 2009), including deciphering the bat virome by our group (Wu et al., 2016). For CoVs, the large RNA genome has a high frequency of mutation and recombination. Conventional molecular diagnosis requires prior knowledge of the target whereas NGST provides an unbiased result. From this point, NGST is more sensitive than PCR, especially for screening RNA virus with a high frequency of variation. However, the high cost, long turn-around time, and sophisticated bioinformatic analysis required, limits the number of samples that can be sequenced. Our mCoV-MS method can be used to complement NGST methods in large-scale screening studies. On one hand, we can screen a large number samples using mCoV-MS method. Then, the selected positive samples or suspicious positive samples were analyzed using the NGST method. On the other hand, new assays can be incorporated into the mCoV-MS method when we obtain new information regarding CoVs, such as the information we get from NGST. The mCoV-MS method is flexible and can be modified as necessary. As an example, we can use the SARS-CoV or MERS-CoV panels on their own.

There are 10 countries in or near the Arabian Peninsula with laboratory-confirmed MERS cases, and 17 countries with travel-associated MERS cases (http://www.cdc.gov/coronavirus/mers/index.html). There is no border for virus. Therefore, nasopharyngeal samples collected from pilgrims have been screened for MERS-CoV (Gautret et al., 2013). Memish et al. stated that their results only represented a relatively small proportion of the total number of pilgrims (Memish et al., 2014). The largest known outbreak of MERS outside the Arabian Peninsula occurred in the Republic of Korea in 2015. The outbreak was associated with a single person returning from the Middle East which involved 16 hospitals and 186 patients (Korea Centers for Disease Control and Prevention, 2015). The WHO encourages all member states to continue their surveillance for acute respiratory infections, in particular SARI (http://www.who.int/csr/don/03-february-2015-mers/en/). The United Kingdom has established a surveillance system to screen people from the Middle East for MERS-CoV (Thomas et al., 2014). Our mCoV-MS method is a suitable choice for this type of screening, as it is possible for one technician to analyze two 384-well plates within 8 h.

We found some limitations of mCoV-MS method just like we mentioned it in previous report (Peng et al., 2013a). When the HCoV load is very low in a sample, the mCoV-MS method may fail to detect it because of the volume of sample used was 2 μl. So we should use a larger primary PCR volume or several primary PCR reactions for those samples. It should be pointed out that our method is based on the known CoVs sequence, as far as possible covers the known CoVs. It is difficult to detect a new HCoV only use mCoV-MS method. However, it can provide some clues for new HCoV detection compared to current methods.

## Ethics statement

This study was carried out in accordance with the recommendations of national ethics regulations and approved by the ethics committee of Institute of Pathogen Biology with written informed consent from all subjects. All subjects gave written informed consent in accordance with the Declaration of Helsinki. The protocol was approved by the ethics committee of Institute of Pathogen Biology.

## Author contributions

JP conceived the experiments, analyzed the results and wrote the manuscript. LX and CZ conducted the experiments and analyzed the results. ZW collected specimens. All authors reviewed the manuscript.

### Conflict of interest statement

Junping Peng, Junhua Guo, and Qi Jin have a patent (ZL201310309972.4, People's Republic of China) related to this report. The other authors declare that the research was conducted in the absence of any commercial or financial relationships that could be construed as a potential conflict of interest.
