# Supplementary material for: Establishment and Application of a Universal Coronavirus Screening Method Using MALDI-TOF Mass Spectrometry
Source: Front Microbiol. 2017 Aug 9;8:1510. doi: 10.3389/fmicb.2017.01510 (PMC5552709; doi:10.3389/fmicb.2017.01510)
Supplement: Supplementary file 5 [file Image5.PDF]

Supplementary Material

Establishment and Application of a Universal Coronavirus Screening Method using MALDI-TOF mass spectrometry

Leshan Xiu+, Chi Zhang+, Zhiqiang Wu, Junping Peng*

* corresponding author: pengjp@hotmail.com

+ these authors contributed equally to this work

Figure S5

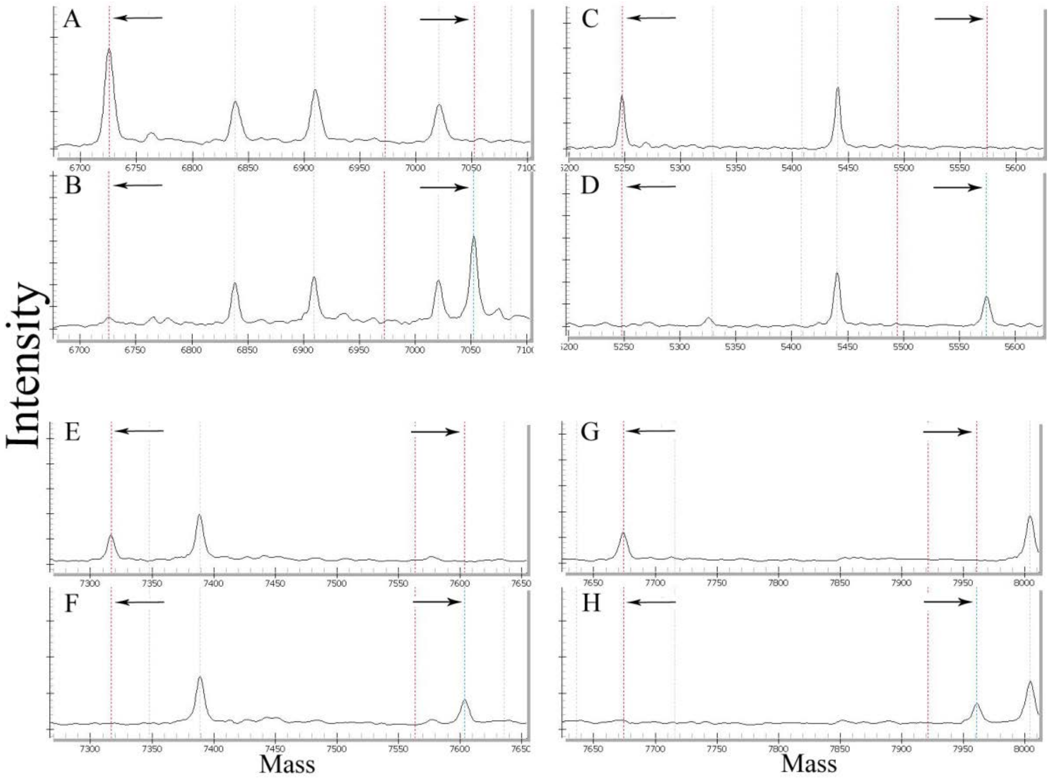

Fig S5 Assessing the specificity of the assay of SARS-CoV using the CoV-MS method. (A) Negative result of *N* assay, (B) Positive result of *N* assay, (C) Negative result of *RdRp* assay, (D) Positive result of *RdRp* assay, (E) Negative result of *ORF1b* assay, (F) Positive result of *ORF1b* assay, (G) Negative result of *upE* assay, (H) Positive result of *upE* assay. In the mass spectrometry mass spectra, the dotted lines in the left and the dotted lines in the right represent the unextended primers and the extended primers of the assay, respectively.
