# Supplementary material for: Establishment and Application of a Universal Coronavirus Screening Method Using MALDI-TOF Mass Spectrometry
Source: Front Microbiol. 2017 Aug 9;8:1510. doi: 10.3389/fmicb.2017.01510 (PMC5552709; doi:10.3389/fmicb.2017.01510)
Supplement: Supplementary file 6 [file Table1.DOCX]

***Supplementary Material***

**Establishment and Application of a Universal Coronavirus Screening Method using MALDI-TOF mass spectrometry**

Leshan Xiu^+^, Chi Zhang^+^, Zhiqiang Wu, Junping Peng*

* corresponding author: pengjp@hotmail.com

^+^ these authors contributed equally to this work

Table S1 Reference strains used in CoV-MS primers design

| **Virus species** | **Strains** | **GenBank accession No.** |
| --- | --- | --- |
| Human coronavirus 229E | 229E | NC_002645 |
|  | 229E/human/USA/932-72/1993 | KF514432 |
|  | 229E/human/USA/933-50/1993 | KF514430 |
|  | 229E/human/USA/933-40/1993 | KF514433 |
|  | J0304 | JX503061 |
|  | 0349 | JX503060 |
| Human coronavirus OC43 | ATCC VR-759 | NC_005147 |
|  | OC43-Paris | AY585229 |
|  | 87309 Belgium 2003 | AY903459 |
|  | HK04-01 | JN129834 |
|  | 2058A/10 | KP198610 |
|  | 1783A/10 | KP198611 |
|  | LY341 | KJ958218 |
|  | OC43/human/USA/851-15/1985 | KF530060 |
|  | OC43/human/USA/971-5/1997 | KF530099 |
|  | OC43/human/USA/911-38/1991 | KF530096 |
| Human coronavirus NL63 | Amsterdam I | NC_005831 |
|  | CBJ123 | JX524171 |
|  | NL63/DEN/2009/9 | JQ765563 |
|  | NL63/DEN/2005/232 | JQ765569 |
|  | NL63/DEN/2008/16 | JQ765566 |
|  | Amsterdam 057 | DQ445911 |
|  | NL63/RPTEC/2004 | JX504050 |
|  | NL63/human/USA/904-20/1990 | KF530104 |
|  | NL63/human/USA/838-9/1983 | KF530110 |
| Human coronavirus HKU1 | HKU1 | NC_006577 |
|  | Human coronavirus HKU1 genotype B | AY884001 |
|  | N19 | DQ415896 |
|  | N20 | DQ415897 |
|  | N25 | DQ415902 |
|  | Caen1 | HM034837 |
|  | HKU1/human/USA/HKU1-5/2009 | KF686340 |
|  | HKU1/human/USA/HKU1-18/2010 | KF430201 |
| SARS coronavirus | GD01 | AY278489 |
|  | WH20 | AY772062 |
|  | ZJ0301 | DQ182595 |
|  | Urbani | AY278741 |
|  | SZ3 | AY304486 |
|  | SZ16 | AY304488 |
|  | BJ01 | AY278488 |
| MERS coronavirus | EMC/2012 | JX869059 |
|  | England-Qatar/2012 | KC667074 |
|  | England 1 | KC164505 |
|  | Munich | KF192507 |
|  | Al-Hasa_1_2013 | KF186567 |
|  | Jordan-N3/2012 | KC776174 |
