# Supplementary material for: Establishment and Application of a Universal Coronavirus Screening Method Using MALDI-TOF Mass Spectrometry
Source: Front Microbiol. 2017 Aug 9;8:1510. doi: 10.3389/fmicb.2017.01510 (PMC5552709; doi:10.3389/fmicb.2017.01510)
Supplement: Supplementary file 7 [file Table2.DOCX]

***Supplementary Material***

**Establishment and Application of a Universal Coronavirus Screening Method using MALDI-TOF mass spectrometry**

Leshan Xiu^+^, Chi Zhang^+^, Zhiqiang Wu, Junping Peng*

* corresponding author: pengjp@hotmail.com

^+^ these authors contributed equally to this work

Table S2 Primers used in the CoV-MS method

| ID | 1^st^ Primer* | 2^nd^ Primer* | Unextended Probe |
| --- | --- | --- | --- |
| **Panel A:** |  |  |  |
| RnaseP | ACGTTGGATGTGAGCGGCTGTCTCCARAAG | ACGTTGGATGAGATTTGGACCTGCGAGRGG | CTTCCGCGCAGAGC |
| MERS-CoV_RdRp | ACGTTGGATGCTAATCGCCAGTACCARCAG | ACGTTGGATGGCCACCRTAGAACTTRGTAG | TCGATGGCTGCAAC |
| MERS-CoV_upE | ACGTTGGATGATGGATTAGCCTCTACRCGG | ACGTTGGATGTCCTCTTCACATAATRGCCC | CTTAAACGCAGAGCTG |
| HKU1_N | ACGTTGGATGGCTAATCACCAAGCTGYCAC | ACGTTGGATGGGAAACCTAGTAGGGARAGC | CTCCGCTGACACTTCTA |
| SARS-CoV_RdRp | ACGTTGGATGAGAGACACTCATAGAYCCTG | ACGTTGGATGGCACTTCTTTCAACTYATGG | GATTGGCCTGTGTTGTA |
| NL63_RdRp | ACGTTGGATGTGAGAACAAGTTTGTRCCTG | ACGTTGGATGTGTCAACCTGTACATTYCCC | TAAACCGTTTGTCCCTGT |
| OC43_RdRp | ACGTTGGATGATGGATGTGGATACACYTCG | ACGTTGGATGGAAGCTACATGCAAAYCTGG | CGTGGATACACATCGTTAT |
| MERS-CoV_ORF1b | ACGTTGGATGATTACGGGAAGCATGYGCAC | ACGTTGGATGCATTACTCGTGAAGAYGCTG | CGCCCATTGAGCACCCTCAA |
| 229E_N | ACGTTGGATGCACTATCAACAAGCAAYGGG | ACGTTGGATGATGGGCTGATGMATCRGAAC | GACACGGGTATTCTACCCTG |
| 229E_RdRp | ACGTTGGATGGAGCGAAGCACAAATCRATC | ACGTTGGATGTTTACTTGGCATGAGYGCAG | ATTACCATCCTGTCTACTAAC |
| SARS-CoV_N | ACGTTGGATGCTGGACCACTATTGGTYTTG | ACGTTGGATGTCTTGGTTCACAGCTRTCAC | CTTGAGGGAATCTAAGTTCCTC |
| MERS-CoV _N | ACGTTGGATGTGATGATCATGGCAACRCTG | ACGTTGGATGTGTAGTTGGGATTCTTRGGG | CCCCCGCCCTGTGTACTTCCTTC |
| OC43_N | ACGTTGGATGGAAGGTCTGCTCCTAYTTCC | ACGTTGGATGATTGCCAGAATTGGCRCTAC | CGCTCCTAATTCCAGATCTACTT |
| SARS-CoV_ORF1b | ACGTTGGATGGACCTTTTTWGAAACGCSCG | ACGTTGGATGATTGACGCTAGCTKGTGCTG | CTGACTTTTAGCTTGTGCTGGTCC |
| NL63_N | ACGTTGGATGATTCCCAGGAATCTTYTCCC | ACGTTGGATGGCCAACGCTCTTGAYCATTC | TTTGGCAGGAATCTTGTCCCTAT |
| HKU1_RdRp | ACGTTGGATGTGCCACATATAGTTCYTAGG | ACGTTGGATGCAATCAGCATACTCARAAAG | AGCATTGGTTAGATCTTTGCTATG |
| SARS-CoV_upE | ACGTTGGATGTAACGTACCTGTTTCTTYCG | ACGTTGGATGTACTAGCGTGCCTTTGYAAG | CTAGAAAGTGCCTTTGTAAGCACAA |
| **Panel B** |  |  |  |
| \| RnaseP \| ACGTTGGATGTGAATAGCCAAGGTGAGCGG \| ACGTTGGATGCGGTGTTTGCAGATTTGGAC \| \| --- \| --- \| --- \| | ACGTTGGATGTGAATAGCCAAGGTGAGCGG | ACGTTGGATGCGGTGTTTGCAGATTTGGAC | CTCCACAAGTCCGC |
| α_1 | ACGTTGGATGCCCATCAATCCAACACTTCC | ACGTTGGATGGTTGCATTGTTCATTGTGCC | ACAAAGGTCCGAAAGC |
| α_2 | ACGTTGGATGAAACAGTGCGTTTATCCACC | ACGTTGGATGCTGAACTCTTACAGTTTGTG | GCGATGACGCAACTATAA |
| β_C | ACGTTGGATGCCCTCTCACAAAGCATGAAG | ACGTTGGATGATCACCACATAGCATGACAG | ACCAGAATGTATTCTGGG |
| α_3 | ACGTTGGATGTACGCTATTTCTGGTAAGGC | ACGTTGGATGGTATTGTCTCGTAGTCATGG | TAGAGCTCGTACAGTAGG |
| β_D1 | ACGTTGGATGACAGGTCCTTAGTGAGATGG | ACGTTGGATGCACACTATTAGCGTATGCAG | AAGGTGGCACTAGTAGTGG |
| α_4 | ACGTTGGATGAGCTTCATCTCCTGCCTTAG | ACGTTGGATGTTGATGTGTTATACCAGTGC | ACAGTTTGTTTTTCTATCGC |
| α_5 | ACGTTGGATGGGAACTCCGTCAATAAACAC | ACGTTGGATGGATTATCACCCAGATTGTGC | TTACACAATGGACCAAAAGC |
| α_6 | ACGTTGGATGAGAGGTTTCTTTGATGAAGG | ACGTTGGATGTCTTTAACAGCAGCATCACC | ATTAAAACATTTCTTCTTCGC |
| α_7 | ACGTTGGATGTGCAAATGTTTAACCCAGTC | ACGTTGGATGCGCTTACCCTTTGTCTAAAC | AAATACTTTACGATACTCAGG |
| α_8 | ACGTTGGATGTAGTCATACCCATGACTGGC | ACGTTGGATGTACCATTGGCATTCCTGGTG | GCATAAGGTACGAATAATAGG |
| β_B | ACGTTGGATGCTGGATACCATTTTCGTGAG | ACGTTGGATGCTGGATCAGCAGCATACACT | TGTACATAATCAGGATGTAAAC |
| α_9 | ACGTTGGATGCATAGTGGGCAGGATGTTAC | ACGTTGGATGGCTGGTCTTTACTATGAGTC | GCTTAGTATAAGCATAAAGTTCA |
| α_10 | ACGTTGGATGCATCCTACCCACTATGACAC | ACGTTGGATGGTTGAGAGGAGTGAAACACC | CTGACACAACTTAATCTTAAGTAT |
| β_D2 | ACGTTGGATGGCGTAAGCATTTTAGTATG | ACGTTGGATGGCCCTGTATATCAGCAACAT | GCATTTTAGTATGATGATCCTTAG |
| α_11 | ACGTTGGATGTTCTCTTCAAGGTATCACAG | ACGTTGGATGGGAGAGATCTTACTGAATAC | TAATTGCGTAAAGCAAAGCACAGG |
| β_A | ACGTTGGATGCCTGGGTAATCAGATCTTGG | ACGTTGGATGCTTGTAAAAGGACTCATCAG | TTGTTATTTTAAGTACTTGTGATGG |

* the specific primers used in this study contained a 5’ 10-base extension (ACGTTGGATG)
